# Supplementary material for: Anomalous Kondo resonance mediated by semiconducting graphene nanoribbons in a molecular heterostructure
Source: Nat Commun. 2017 Oct 16;8:946. doi: 10.1038/s41467-017-00881-1 (PMC5643342; doi:10.1038/s41467-017-00881-1)
Supplement: Supplementary file 1 — Supplementary Information [file 41467_2017_881_MOESM1_ESM.pdf]

Supplementary Figures

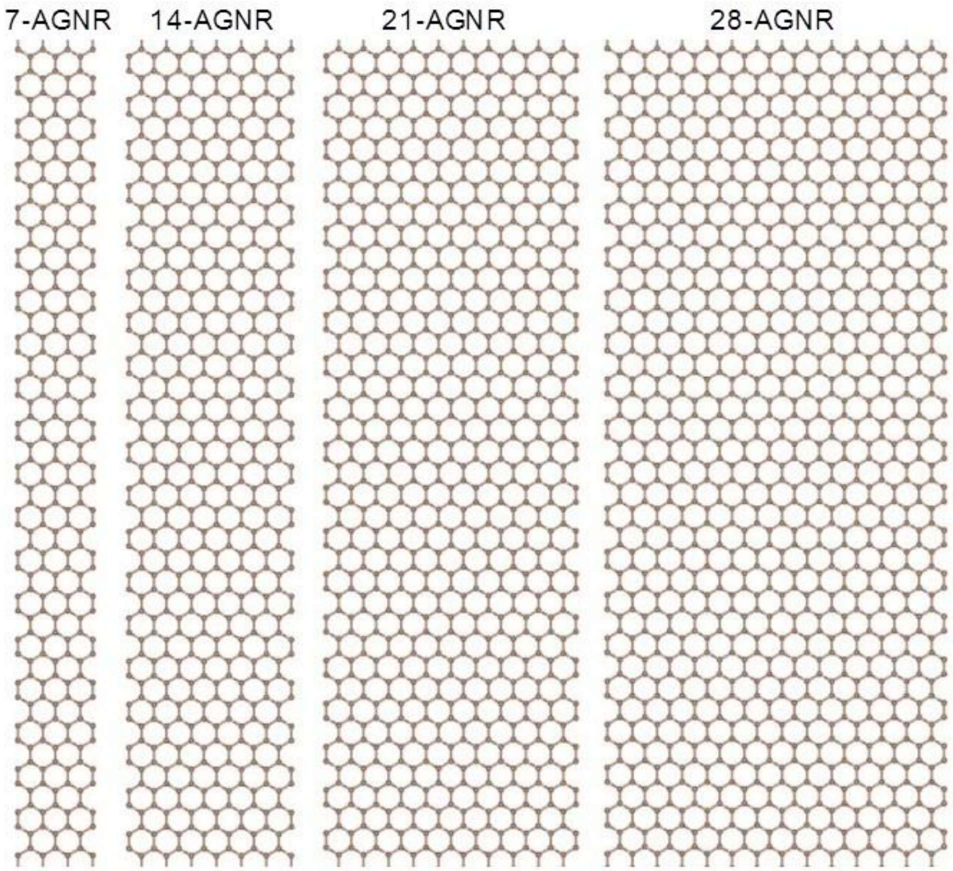

Supplementary Figure 1 | Models of 7-AGNR, 14-AGNR, 21-AGNR, 28-AGNR.

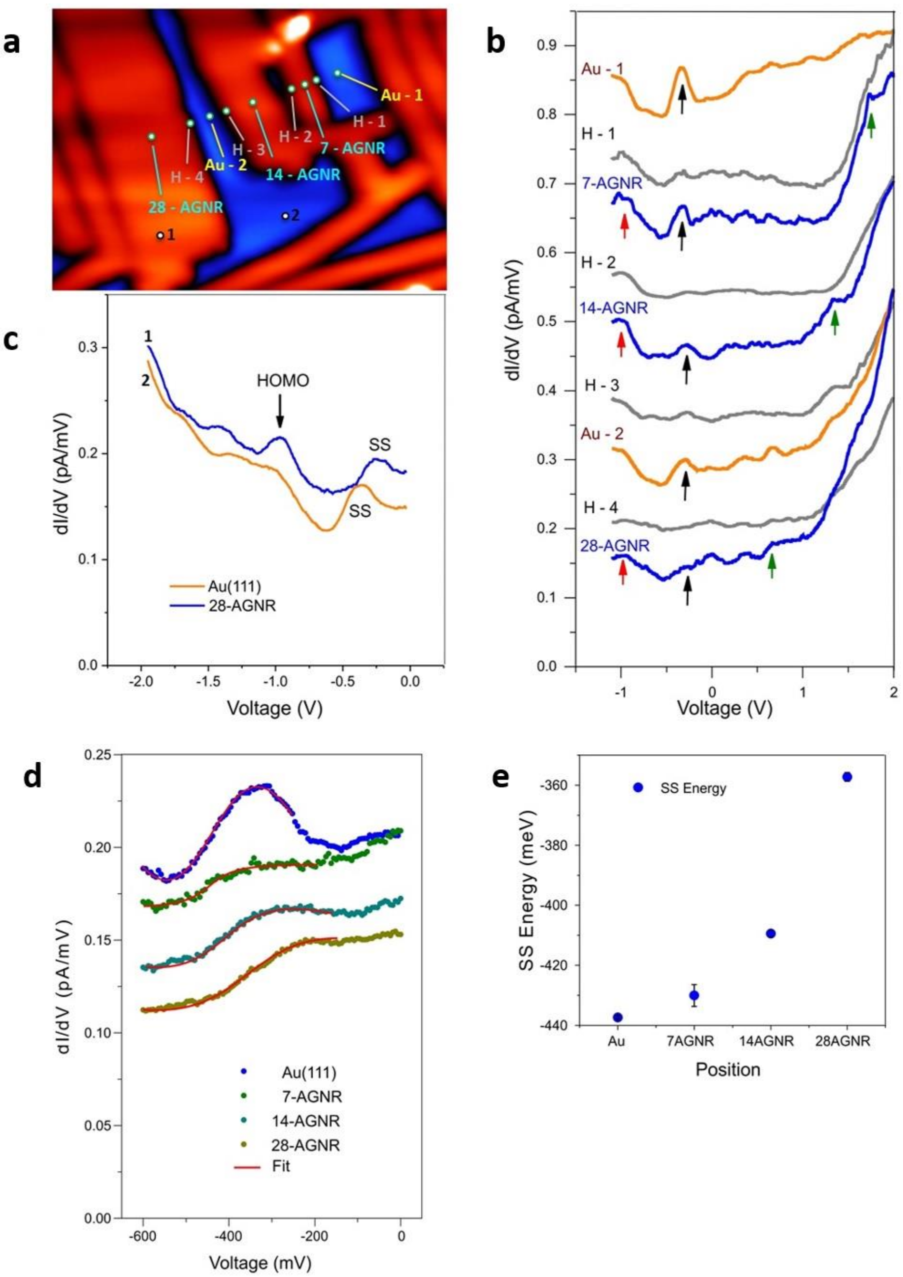

Supplementary Figure 2 | **a**, An STM image of AGNRs with dots showing the location of dI/dV spectroscopy measurements [13.8 x 10 nm^2, I_t = 1 x 10^{-10} A, V_t = 1 V]**. b,** Large bias range dI/dV data measured on single points on Au(111), H-edge of AGNR, and 7, 14, and 28 AGNR**s** corresponding to the locations shown in '**a**' [Tip set-point: I_0 = 1.0 x 10^{-10}A, V_0 = 1.0 V. Spectra are vertically shifted by 0.15 pA/mV for clarity]. **c,** Large bias range dI/dV spectra of AGNR and Au(111) surface (indicated with the location 1, and 2 in '**a**') measured between -2.0 V to 0V. **d**, dI/dV spectra showing surface state measured on Au(111) and 7-,14- and 28-AGNRs. Each spectrum is an average of ~20 to ~80 data measured across the width of respective AGNRs. [Tip set-point: I_0 = 1.0 x 10^{-10}A, V_0 = 1.0 V. Spectra are vertically shifted by 0.05 pA/mV for clarity]. **e**, Surface state onset energy shift towards the Fermi level (0 V) by increasing the width of AGNRs. The error bars represent 95% confident interval of fitted result.

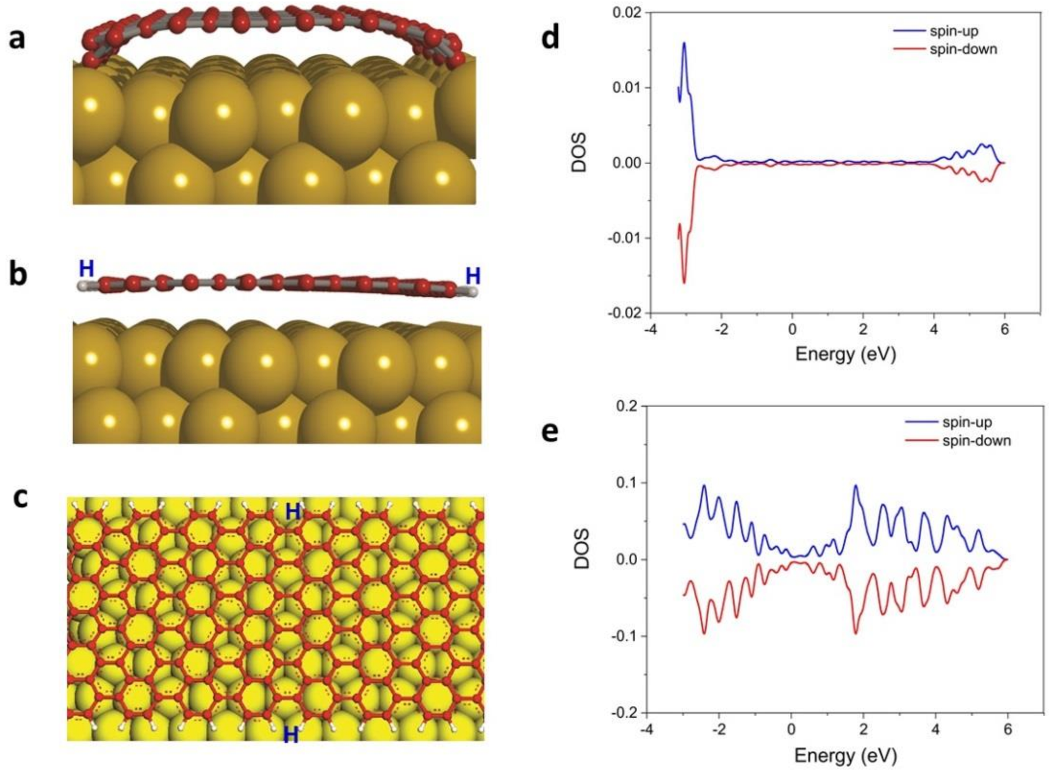

Supplementary Figure 3 | a, DFT calculations reveal that the AGNR binds to the Au(111) surface atoms and forms a bent structure. Surface Au atoms underneath the edges are also buckled upward for binding the AGNR. **b**, Side view of H terminated AGNR. When the edges are terminated by H atoms, the AGNR remains flat on the surface. **c**, Top view of (**b**). **d**, Calculated PDOS at the H atoms shows a large gap. **e**, Calculated PDOS of a C atom at the centre of AGNR.

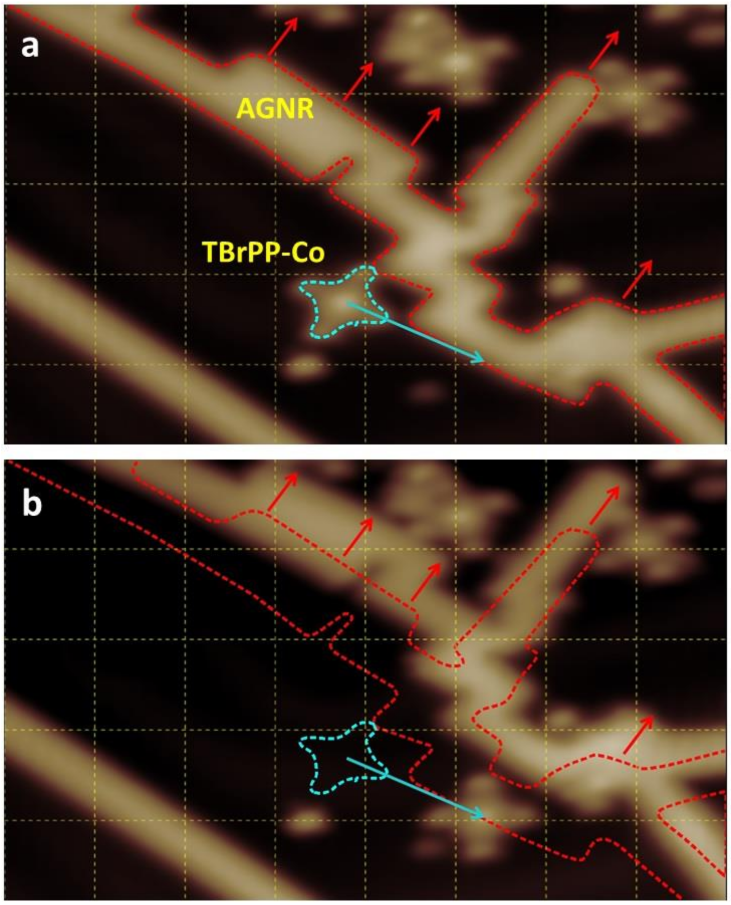

Supplementary Figure 4 | a, An STM image before manipulation [16 x 10 nm^2 , I_t = 1.0 x 10^{-10} A, V_t= 1 V]. When the surface is not fully covered by AGNRs, TBrPP-Co molecules prefer to adsorb on the bare Au(111) surface areas, rather than on top of the AGNRs. We have attempted to position a TBrPP-Co molecule onto a neighbouring AGNR by means of STM tip manipulation A blue arrow indicates the path and direction of manipulation, and the red arrows indicate the direction of AGNR network movement direction. Manipulation parameters: [V_{LM} = 0.1V, R_{LM} = 1 x 10^6 Ω]. **b**, The STM image after manipulation [16 x 10 nm^2, I_t = 1.0 x 10^{-10} A, V_t = 1 V] shows that the TBrPP-Co molecule pushes away the whole AGNR network, instead of moving on top of the AGNR confirming a very weak interaction between AGNR and Au(111) surface as well as preferential positioning of the TBrPP-Co on Au(111). A 2nm x 2nm grid is drawn for eye guidance. The red dashed area shows the initial AGNR network position while the blue dashed area marks the initial TBrPP-Co molecule position.

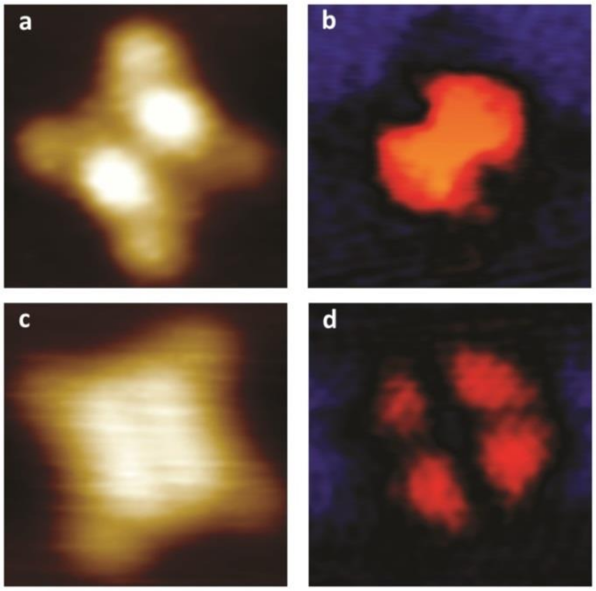

Supplementary Figure 5. The HOMO and LUMO shapes of the TBrPP-Co on Au(111) are determined by means of dI/dV maps. **a**, An STM image of TBrPP-Co on Au(111) acquired at -1V [2.4 x 2.4 nm^2, I_t = 1 x 10^{-10} A]. **b**, Corresponding dI/dV map measured at -1V showing a two-lobe shape of HOMO [Tip set-point: I_0 = 1.0 x 10^{-10}A, V_0 = -1.0 V]. c, An STM image of TBrPP-Co on Au(111) acquired at +2V [2.4 x 2.4 nm^2, I_t = 1 x 10^{-10} A]. **b**, Corresponding dI/dV map measured at +2V showing a four-lobe shape of LUMO [Tip set-point: I_0 = 1.0 x 10^{-10}A, V_0 = +2.0 V].

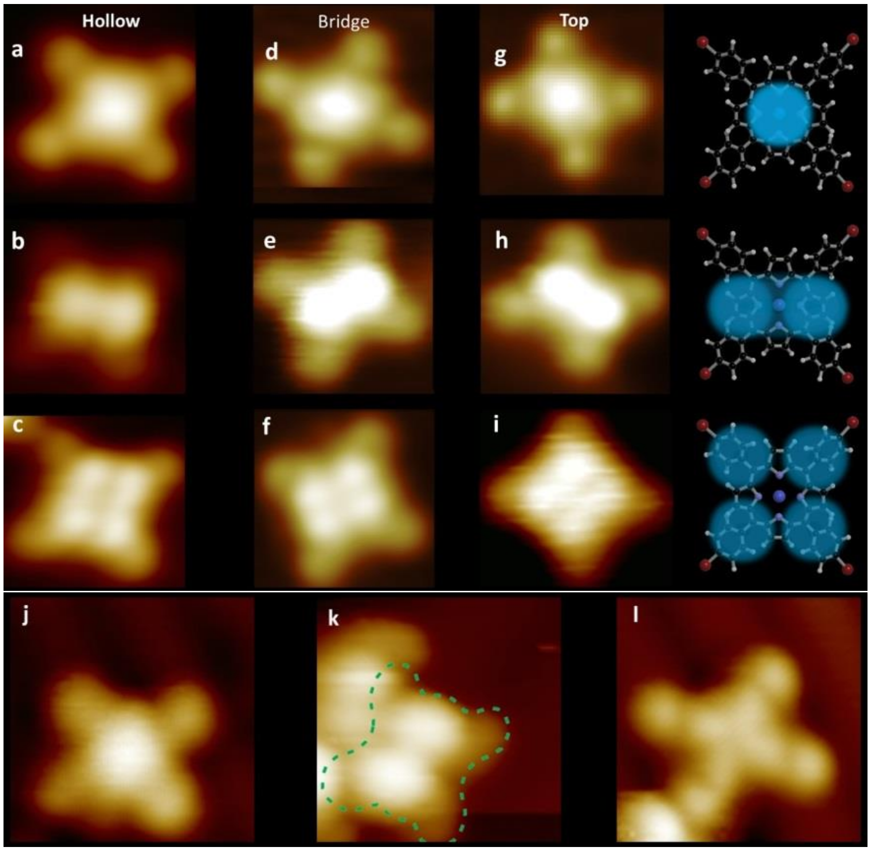

Supplementary Figure 6. STM images of TBrPP-Co on Au(111) recorded as +1 V (**a, d, g**), -1 V (**b,e,h**)), and +2V (**c,f,i**)). [2.4 x 2.4 nm^2, I_t = 2 x 10^{-10} A] for the hollow, bridge and top adsorption sites on Au(111) surface exhibit one, two and four-lobe structures, respectively. The drawings at right show the location of respective lobes in the molecules. **j,k,l,** STM images of TBrPP-Co on AGNRs [2.9 x 2.9 nm^2, V_t = +1V, -1V and +2V respectively, I_t = 1 x 10^{-11} A].

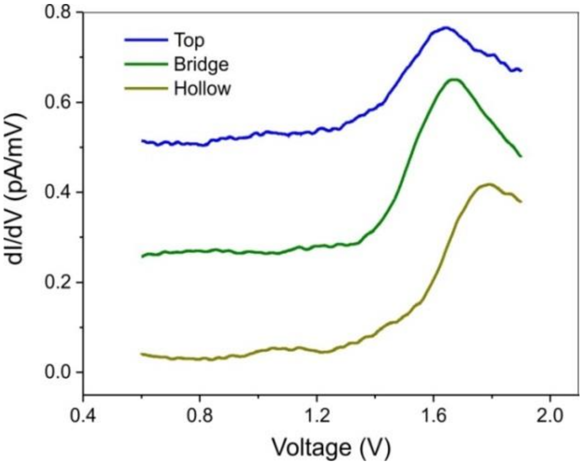

Supplementary Figure 7 | dI/dV-V tunneling spectroscopy data of TBrPP-Co adsorbed on top, bridge, and hollow sites on Au(111) reveal slight variations in LUMO energies. [Tip set-point $I_0 = 1.0 \times 10^{-10}$ A, V_t = -2 V. Spectra are offset by 0.25 pA/mV for clarity].

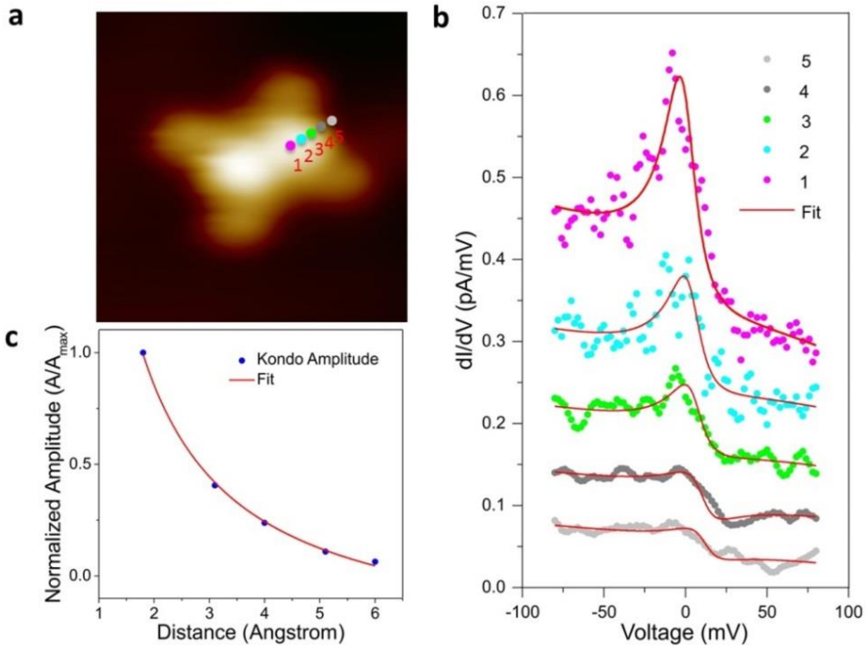

Supplementary Figure 8 | a, An STM image of TBrPP-Co adsorbed on Au(111) surface [2.6 x 2.6 nm^2, I_t = 5.0 x 10^{-11} A, V_t = -1 V]. **b**, A sequence of dI/dV spectra taken at the locations indicated with coloured dots labelled from 1 to 5 in '**a**' show decreasing Kondo amplitudes as the tip moves away from the molecule centre [tip set-point: I_0 = 5.0 x 10^{-11}A, V_0 = 1V]. This TBrPP-Co is adsorbed on a bridge site on Au(111), and the resultant Kondo temperature in this measurement reveals T_K of 135 K for all the curves. Spectra are vertically shifted by 0.8 pA/mV for clarity. **c**, The normalized Kondo amplitude (A/A_{max}) as a function of distance 'r' from the molecule centre shows expected 1/r behaviour, where 'r' is the distance from the centre of the molecule. The Kondo amplitudes are normalized to the maximum amplitude measured at the centre of the Co atom of the molecule.

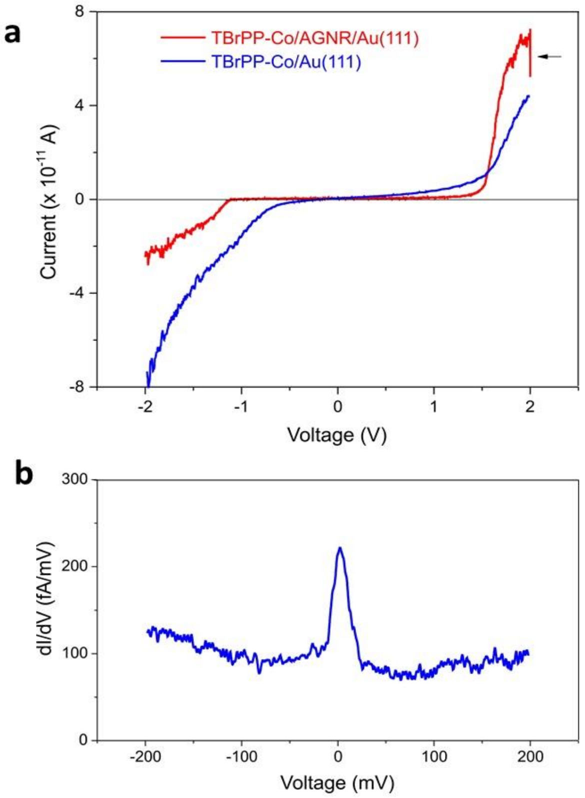

Supplementary Figure 9 | a, I-V tunnelling spectroscopy of TBrPP-Co on AGNR (red) reveals almost zero slope in the gap region. The base current here is ~10 fA, which is the noise level of our lock-in amplifier. This spectrum was simultaneously measured with the dI/dV-V tunnelling spectroscopy plot shown in Fig. 3c. The vertical line at the right end of the signal was caused by the retraction of the tip at the end of the spectroscopic measurement (marked with an arrow). [Tip set-point: $I_0 = 1.0 \times 10^{-11}$A, V_0 = 1.99 V]. In contrast, the I-V tunnelling spectroscopy data of TBrPP-Co directly adsorbed on Au(111) surface (the blue curve), shows a small slope in the gap region near the surface Fermi level [Tip set-point: $I_0 = 5. \times 10^{-11}$A, V_0 = -1.0V]. **b**, A high resolution dI/dV spectrum measured over ± 200 meV bias range shows a Kondo peak at the Fermi level. [Tip set-point: $I_0 = 1.0 \times 10^{-11}$A, V_0 = 0.2 V].

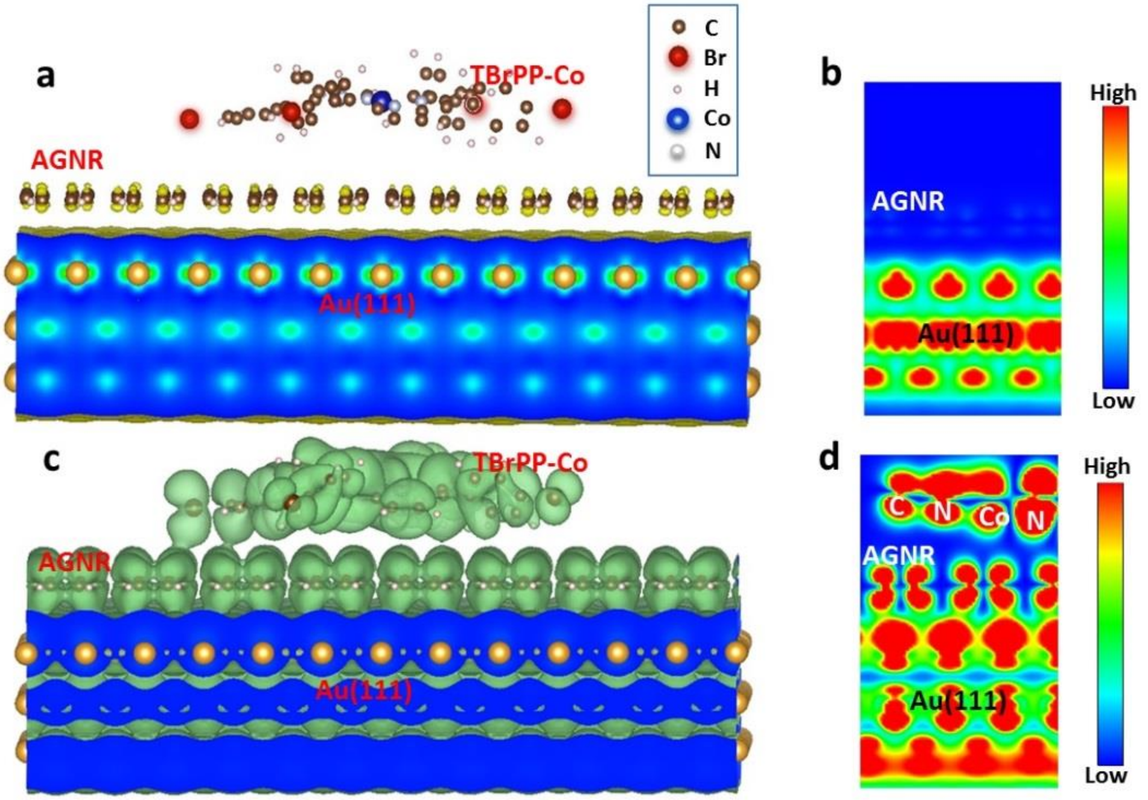

Supplementary Figure 10 | a, Calculated charge densities (integrated from -0.5 eV to 0.5 eV) of TBrPP-Co/AGNR/Au(111) heterostructure within the AGNR band gap reveal that the semiconducting AGNR behaves as an insulator and electronically decouples the TBrPP-Co molecule and the Au(111) surface underneath. **b**, A cross-sectional charge density plot of '**a**' shows no coupling between the TBrPP-Co, AGNR and Au(111) within the bandgap. **c**, Calculated charge densities of TBrPP-Co/AGNR/Au(111) heterostructure beyond the AGNR band gap-edge (integrated from -1 eV to 1 eV) showing electronic coupling of the orbitals; Now the C 'p_z' orbitals of the AGNR are clearly coupled to the Au(111) DOS underneath. **d**, Corresponding cross-sectional plot of '**c**' showing the N 'p', Co 'd' in the molecule as well as C 'p_z' and Au 's' orbitals. Here Co density overlaps with N atoms. The positions of Co, N, C in AGNRs and Au atoms are marked for clarity.

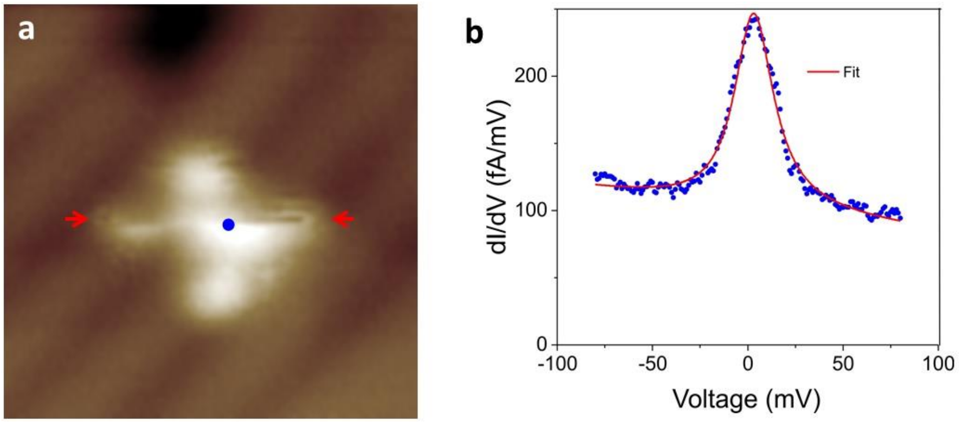

Supplementary Figure 11 | a, An STM image shows a TBrPP-Co molecule adsorbed on top of a 7AGNR [4.5 x 4.5 nm^2, I_t = 3.0 x 10^{-12} A, V_t = 0.5 V]. Even with a small tunnelling current (I_T = 3pA), a TBrPP-Co molecule is moved by the STM tip to the centre of a 7-AGNR during scanning, which is indicated by two red arrows. **b**, A dI/dV spectrum taken at the location indicated with a blue dot in **a** reveals a Kondo peak [T_K = 144.8 K, q = 39.7] around the Fermi level [tip set-point: I_0 = 3.0 x 10^{-12} A, V_0 = 0.5 V]. The Kondo temperature of the molecule on 7-AGNR, T_K = 144.8 K, is about the same as the ones observed for top Au(111) surface site on 21-AGNR.

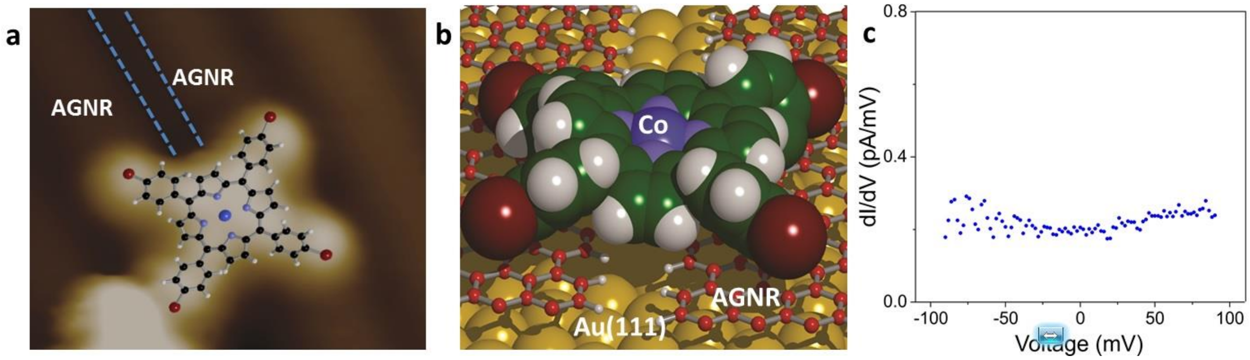

Supplementary Figure 12. **a**, An STM image of TBrPP-Co adsorbs between two AGNRs [3.5 x 3.1 nm^2, I_t = 1.0 x 10^{-11} A, V_t = 0.5 V]. Here, the TBrPP-Co molecule adsorbs at a bridge position between two AGNRs with its centre located directly above Au(111) surface at a vertical distance of 7.5Å. **b**, The corresponding model. **c**, A dI/dV tunneling spectrum measured at the central part of the molecule does not shows Kondo resonance [tip set-point: I_0 = 1.0 x 10^{-11} A, V_0 = 0.5 V]. This further confirms that the AGNR is key in mediating the observed Kondo effect.

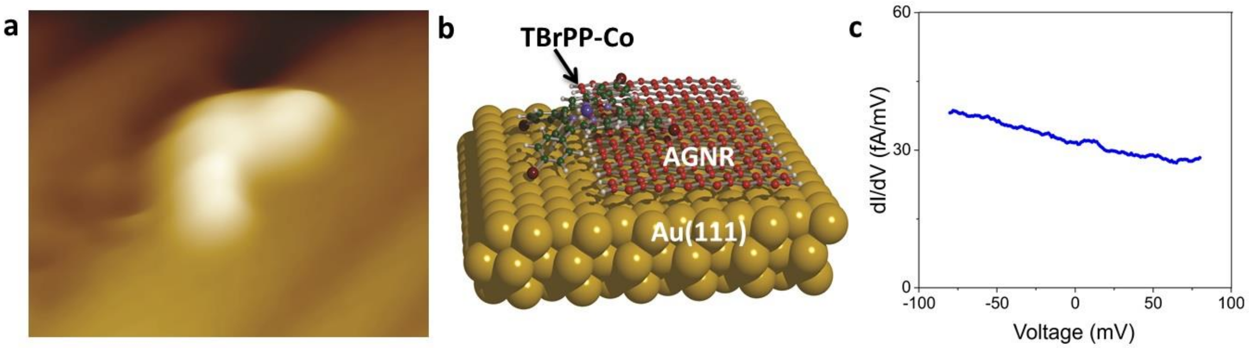

Supplementary Figure 13 | a, An STM image of TBrPP-Co adsorbed at the edge of an AGNR [4.0 x 3.2 nm^2, I_t = 5.0 x 10^{-12} A, V_t = 0.1 V]. Here, two of the bromo-phenyl legs of TBrPP-Co are positioned on top of AGNR while the other two legs are directly adsorbed on the Au(111) surface. In this position, the central Co atom of the molecule is directly located at the AGNR edge. **b**, A corresponding model. **c**, dI/dV tunnelling spectrum measured at the central part of the molecule does not show a Kondo resonance [tip set-point: I_0 = 5.0 x 10^{-12} A, V_0 = 0.1 V].

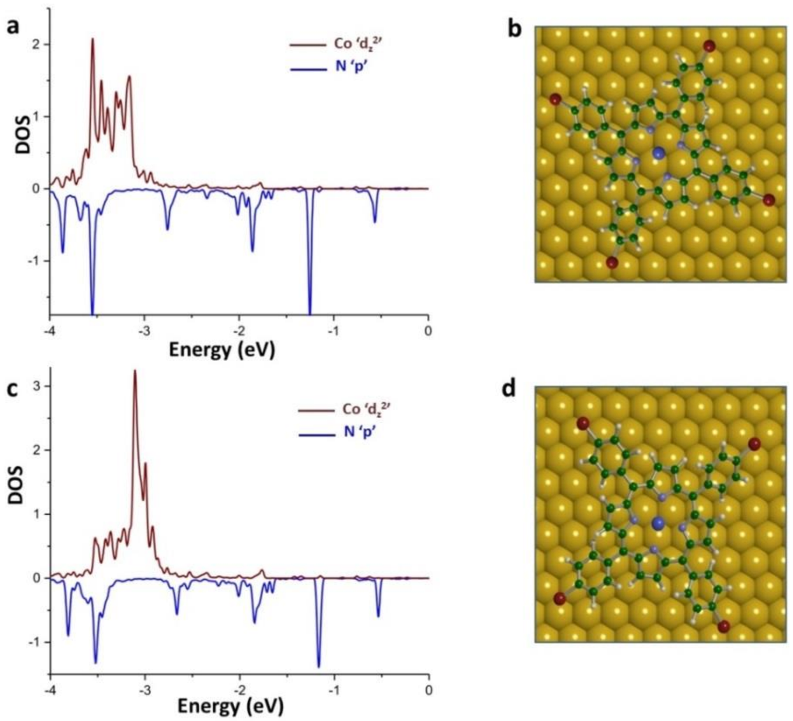

Supplementary Figure 14 | a, SP-PDOS of Co 'd_z^2' and N 'p' orbitals for the TBrPP-Co adsorbed on a bridge site. **b**, Corresponding adsorption model calculated by DFT+U. **c**, SP-PDOS of Co 'd_z^2' and N 'p' orbitals for the TBrPP-Co adsorbed on a hollow site. **d**, Corresponding adsorption model as calculated by DFT+U.

Supplementary Notes

Supplementary Note 1

The electronic structures of AGNRs are measured by means of large bias range dI/dV spectra. Supplementary Fig. 2a and 2b show an STM image of AGNRs marking the locations of dI/dV point spectroscopy and the corresponding spectra. In order to capture the LUMO orbital of the AGNRs, the point spectra were taken between -1 V to +2V range (Supplementary Fig. 2b). The HOMO of AGNR is measured at -0.96 V (Supplementary Fig. 2c), which is in agreement with previous measurements [Ref. 4, 23]. The HOMO level remains the same for 7, 14, and 28 AGNRs. The LUMO level, however, is reduced for increasing widths of AGNRs, as expected. For 7-AGNR, the HOMO-LUMO gap is measured as ~2.7 eV, which is similar to the reported values in the literature [4, 23]. For 14 and 28-AGNRs, the HOMO-LUMO gaps are determined as ~2.3eV and ~1.6 eV, respectively (Supplementary Fig. 2b).

In Supplementary Figure 2b, the dI/dV spectrum measured on Au(111) surface (Au-1) shows a cogent surface state (SS) of Au(111) while the one measured in between two AGNRs (Au-2) exhibits a reduced Au intensity presumably due to screening of the two neighbouring AGNRs. The dI/dV spectra of the H terminated edges (H-1, H-2, H-3, and H-4) reveal reduced dI/dV intensities due to the blocking by H atoms to the tunnelling process; they appear to follow the spectroscopic features of the corresponding AGNRs. Although the tip is positioned on top of the H-terminated edge, tunnelling still involves the neighbouring C atoms from AGNR, and the Au(111) surface underneath. Thus, the dI/dV spectra of H-terminated AGNR edges reveal convolutions of the respective AGNR and the Au(111) surface structures. For instance, the spectrum measured at the 7-AGNR edge (H-1) reveals a rise in dI/dV signal ~+1.2 V, which is also observed in 7-AGNR but not on Au(111) surface (Au-1). The spectrum measured at the opposite edge of 7-AGNR (H-2) shows much reduced intensity than H-1 probably due to screening by the nearby 14-AGNR. Similarly, the spectra H-3 and H-4 approximately follow the features observed on 14- and 28-AGNRs, respectively. The dI/dV spectra of AGNRs also reveal a convolution of Au(111) states and the HOMO and LUMO gap of AGNRs. Although the AGNRs have bandgaps, the tunnelling process occurs ultimately between the STM tip and the conducting Au(111) surface underneath the AGNR, as provided by Au states located in the AGNR bandgaps. Once the HOMO and LUMO edges of AGNR are reached then the dI/dV signal intensity increases because now AGNR states are involved in the tunnelling process.

In Supplementary Figure 2d and 2e, the shift of Au(111) SS onset energy is examined using dI/dV tunnelling spectroscopy on top of bare Au(111) surface, and 7-, 14- and 28-AGNRs on Au(111) surface. SS energies are extracted from the dI/dV spectra using the following fitting equation[1]:

$$\frac{dI}{dV} = C + A\int_{-\infty}^{\infty} \frac{\Sigma}{E^2 + \Sigma^2} f(E - V_b + E_s)dE$$

where ‘C’ and ‘A’ are constants related to bulk and surface state densities, ‘ Σ ’ is the self-energy related to broadening, ‘ E ’ is an independent variable, ‘V_b’ is the bias, and ‘ E_s ’ is the surface state energy.

Resultant value of bare Au(111) surface state energy -437.3meV (at T=12K) is in agreement with previous measurements[2,3,4,5]. Underneath the AGNRs, the surface state energy shifts towards higher energies with increasing width of AGNR (Supplementary Fig. 2b).

Supplementary Note 2

13- AGNR is used in the calculations for Supplementary Fig. 3, it exhibits a corresponding band-gap. The PDOS here are calculated for 3.3Å above the Au(111) surface. The PDOS of the H edge shows a large gap in accordance with the measured dI/dV-V spectroscopy shown in Fig. 1c and Supplementary Fig. 2b. H atoms for the termination of AGNR edges are available on the surface synthesis procedure used in this experiment because the formation of AGNRs from the polymer chain involves breaking C-H bonds.

Supplementary Note 3

The TBrPP-Co molecules appear as two-lobe or four-lobe structures when the bias exceeds the corresponding orbital energies independent of the adsorption sites (Supplementary Fig. 5, and Fig. 6). When images are taken with the biases within the HOMO-LUMO gap, the molecule appears as a single protrusion at the centre for top, bridge, and hollow sites on Au(111). At ~-1V, which exceeds the energy of the HOMO (Fig. 2g in the text), the molecule appears as a two-lobe shape for all three surface sites. At ~+2V, which is close to the energy of the LUMO, the molecule appears as a four-lobe structure for all three surface sites. Similarly one-lobe, two-lobe and four-lobe shapes of the molecules are also observed on AGNR. The two-lobe and 4-lobe structures for the HOMO and LUMO shapes of the molecule have been observed previously on Cu(111) surface[6] as well.

Supplementary Note 4

The Kondo resonances are fitted using the following formula[7]**;**

$\frac{dI}{dV} \propto \frac{q(r)^2+2q(r)\varepsilon-1}{1+\varepsilon^2}$, $\varepsilon = \frac{meV+\Delta E}{k_B T_K}$, and $A(r) \propto [1+q(r)^2]$

Where *A(r)* is the Kondo amplitude, and *r* is the distance from the centre of the molecule.

Supplementary Note 5

Kondo temperatures (T_K) for the molecules on Au(111) surface are slightly dependent on the adsorption sites. The observed T_K values exhibit a decreasing trend from the top (150K) to the bridge (130.7 K) and hollow (112.4 K) sites. Consequently, LUMO orbital energies vary from 1.64 V for the top site, to 1.7 V for the bridge site, and 1.8 V for the hollow sites (Supplementary Fig. 7) while the HOMO for all three sites remains the same at ~-0.9 V. Thus, the HOMO-LUMO gaps of TBrPP-Co are 2.54 V for the top site, 2.6 V for the bridge site, and 2.7 V for the hollow site, respectively. Here, the smaller the bandgap the stronger the molecule surface interaction and therefore the molecule-surface interaction is the strongest for the top site followed by the bridge and hollow sites. Thus, the molecule-surface interaction strengths follow the same trend as the Kondo temperature. Similar behaviour was also reported for the spin

exchange coupling strength of Co atoms adsorbed on a CuN insulating layer by the group of Hirjibehedin[8].

Supplementary Note 6

Calculated spin polarized projected DOS of Co 'd_{z^2}' and N 'p' orbitals for the TBrPP-Co adsorbed on a bridge site and hollow site are rather similar to the ones obtained for the top site (Fig. 4c in the text, and Supplementary Fig. 14). The broadening of the Co 'd_{z^2}' orbital due to hybridization is slightly different in the bridge, top, and hollow sites. This difference is then reflected in the Γ values used in Eq. 1 to calculate respective Kondo temperatures (T_K), and extracted from these results. In the multipeak PDOS of the Co d_{z^2} shown in the figures for the different environments, the Γ values are calculated by considering the average of the different peak widths, weighted by their relative amplitude.

Supplementary Note 7

Spin sensitive DFT calculations of TBrPP-Co adsorbed on Au(111) and on AGNR/Au(111) reveal that although N atoms have a much smaller magnetic moment as compared to the central Co atom, the spins of the four N atoms align in opposite direction of Co spin.

TBrPP-Co/Au(111)

s	p	d	Total magnetic moment	Atom
-0.0003	-0.013	0.00	-0.016	N
-0.0003	-0.013	0.00	-0.016	N
-0.0003	-0.013	0.00	-0.016	N
-0.0003	-0.013	0.00	-0.016	N
0.028	0.006	1.007	1.041	Co

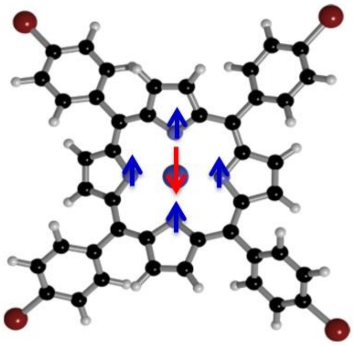

TBrPP-Co/AGNR/Au(111)

S	p	d	Total magnetic moment	Atom
0.003	0.013	0.00	0.016	N
0.003	0.013	0.00	0.016	N
0.003	0.013	0.00	0.016	N
0.003	0.014	0.00	0.017	N
-0.029	-0.006	1.007	-1.014	Co

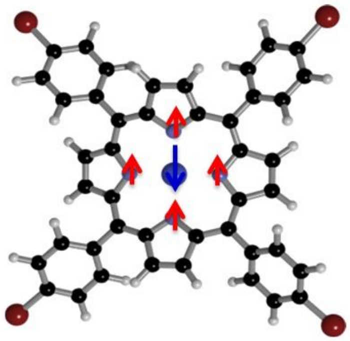

Supplementary Table 1.

Ad-site	$d_z{}^2$ state	T_K
**Top**	69.4 %	150.0 K
**Bridge**	65.9 %	130.7 K
**Hollow**	63.9 %	112.4 K

To further probe the effect of adsorption site on the resulting Kondo temperatures, T_K, we have analysed the calculated PDOS of $d_z{}^2$ orbital for the molecule adsorbed on the three adsorption sites on Au(111) shown in Fig. 4c in the main text, and Supplementary Fig. 14. The strengths of molecule surface interactions and corresponding T_K values are found proportional to the available $d_z{}^2$ electronic state of each adsorption site configuration. This figure of merit is acquired by integrating the PDOS in each case over a fixed energy window, from -4eV to the Fermi level (0eV). The highest weight of the $d_z{}^2$ orbital is found for the top site while the hollow site has the lowest. Thus, the integrated PDOS of $d_z{}^2$ orbital also follow the trend of the observed T_K values.

Supplementary References

1 Li, J., Schneider, W.-D., Berndt, R., Bryant, O.R. & Crampin, S. *Phys. Rev. Lett.* **81,** 4464–4467 (1998).

2 Kevan, S.D. & Gaylord, R.H. *Phys. Rev. B* **36,** 5809–5818 (1987).

3 Andreev, T., Barke, I. & Hövel, H. *Phys. Rev. B* **70,** 205426 (2004).

4 Paniago, R., Matzdorf, R., Meister, G. & Goldmann, A. *Surf. Sci.* **336,** 113–122 (1995).

5 Hansson, G.V. & Flodström, S.A. *Phys. Rev. B* **18,** 1572–1585 (1978).

6 Iancu, V. PhD Dissertation, Ohio University (2006).

7 Újsághy, O., Kroha, J., Szunyogh, L. & Zawadowski, A. *Phys. Rev. Lett.* **85,** 2557-2560 (2000).

8 Oberg, J.C., Calvo, M.R., Delgado, F., Moro-Lagares, M., Serrate, D., Jacob, D., Fernandez-Rossier, J., & Hirjibehedin, C.F. *Nature Natech.* **9**, 64-68 (2014).
