# Supplementary material for: Anomalous Kondo resonance mediated by semiconducting graphene nanoribbons in a molecular heterostructure
Source: Nat Commun. 2017 Oct 16;8:946. doi: 10.1038/s41467-017-00881-1 (PMC5643342; doi:10.1038/s41467-017-00881-1)
Supplement: Supplementary file 3 — Description of Additional Supplementary Files [file 41467_2017_881_MOESM3_ESM.pdf]

Description of Additional Supplementary Files

File Name: Supplementary Movie 1
Description: A movie of two-dimensional tunneling spectroscopic maps acquired from 300 mV to -300 mV bias range revealing Kondo signature.
